# Clinical Evidence on Expansion of Essential Oil-Based Formulation’s Pharmacological Activity in Bovine Mastitis Treatment: Antifungal Potential as Added Value

**DOI:** 10.3390/antibiotics13070575

**Published:** 2024-06-22

**Authors:** Dragana Tomanić, Dragana D. Božić, Nebojša Kladar, Marko Samardžija, Jelena Apić, Jovan Baljak, Zorana Kovačević

**Affiliations:** 1 Department of Veterinary Medicine, Faculty of Agriculture, University of Novi Sad, Trg Dositeja Obradovica 8, 21000 Novi Sad, Serbia; 2 Department of Microbiology and Immunology, Faculty of Pharmacy, University of Belgrade, Vojvode Stepe 450, 11221 Belgrade, Serbia; 3 Center for Medical and Pharmaceutical Investigations and Quality Control, Department of Pharmacy, Faculty of Medicine, University of Novi Sad, Hajduk Veljkova 3, 21000 Novi Sad, Serbiajovan.baljak@mf.uns.ac.rs (J.B.); 4 Department of Pharmacy, Faculty of Medicine, University of Novi Sad, Hajduk Veljkova 3, 21000 Novi Sad, Serbia; 5 Faculty of Veterinary Medicine, University of Zagreb, Heinzelova 55, 10000 Zagreb, Croatia; 6 Scientific Veterinary Institute Novi Sad, Rumenački put 20, 21000 Novi Sad, Serbia

**Keywords:** essential oils, ethno-veterinary, yeast, mastitis, Phyto-Bomat

## Abstract

Bovine mastitis, as a significant and prevalent health problem in dairy herds, is primarily attributed to bacterial infections. Yeasts, although considered relatively rare causative agents, have also been associated with mastitis in dairy cattle. Current mastitis treatment predominantly relies on antibiotics, with limited emphasis on antifungal treatment. However, mycotic mastitis is challenging to treat, since these fungi are often resistant to antibiotics and may even utilize them for energy. In the current research, the in vivo antimicrobial activity of the essential oil-based formulation (Phyto-Bomat), as a possible alternative mastitis treatment associated with yeasts, was studied. This study involved a total of 68 animals from two dairy farms with diagnosed mastitis, and three treatment groups were established: conventional antibiotic treatment, Phyto-Bomat treatment, and the combination of both. The findings suggest significant variations in the presence of Candida samples based on the treatment administered, and the most significant difference was noted in cows treated with the combination (Phyto-Bomat and antibiotics). Yet, it is important to note that the results reveal that, regardless of the treatment type, there are statistically significant differences in the presence of Candida samples across the examined time points. These results aim to provide valuable insights into the potential of EOs as an alternative therapy in bovine mastitis, specifically targeting yeasts. Such findings could offer new strategies in the mycotic mastitis control and reducing the occurrence of secondary infections following antibiotic treatment.

## 1. Introduction

Inflammation of the mammary gland is the most important health problem in bovine dairy herds [1]. Bovine mastitis is a multifactorial disease, regarded as the most prevalent and economically significant infectious condition affecting dairy cattle globally [2]. It is estimated to result in annual losses of approximately USD 35 billion in the dairy industry [3]. The etiology of bovine mastitis involves a wide range of microorganisms, mainly comprised of bacteria, viruses, mycoplasma, algae, and yeasts [2,4,5]. Bacteria are typically recognized as the primary pathogens in most cases of mastitis, with yeasts generally considered as secondary mastitis-causing agents. Despite their limited pathogenicity, yeasts can cause various infections in both humans and domestic animals [6].

Yeasts are considered as one of the bovine mastitis causative agents with increasing incidence observed lately [6,7,8,9]. Different fungal species have been associated with mastitis in dairy cattle, such as the yeasts *Candida* spp., *Cryptococcus neoformans*, and *Trichosporon* spp.; the molds *Aspergillus fumigatus*, *Cephalosporium* spp., and *Mucor* spp.; and dimorphic fungi like *Coccidioides* spp. and *Histoplasma* spp. [7,10]. Moreover, fungal infections of the mammary gland are predominantly caused by yeasts of the *Candida* genus [1,9], with the most reported species being *Candida albicans*, *Candida krusei*, *Candida rugosa*, and *Candida guilliermondii* [7].

Mycotic mammary infections can be sporadic or outbreak-related, with severity determined by yeast quantity and species [11]. Compromised immune system of the host, imbalance of bacterial microflora, and particularly uncontrolled and excessive use of antibiotics may predispose glands to yeast infections [6,10]. Moreover, high doses of antibiotics can promote the growth of yeast, particularly *Candida* spp., as they can use penicillin and tetracycline as a source of nitrates [12]. The presence of yeasts in the mammary gland typically results in a subclinical form of the infection, which is mainly characterized by an increased somatic cell count [11]. Furthermore, clinical form is manifested by udder swelling, pain, and dramatically reduced milk production [1]. Rarely, they have the potential to lead to fatal consequences [6], while in recent years, higher morbidity rates caused by mycotic mastitis in cattle have been reported [13].

Routine mastitis treatment involves predominantly antibiotics, while much emphasis is not given on the antifungal therapy [8]. Additionally, mycotic mastitis treatment is challenging because these fungi often resist antibiotics and even utilize them for energy [12]. Moreover, despite promising in vitro activity, antifungal agents commonly used in clinical settings, such as Amphotericin B, Nystatin, Itraconazole, Clotrimazole, Fluconazole, or Voriconazole, can be toxic to mammary tissue, potentially causing harm in mycotic mastitis treatment [12,14]. Hence, most of the mastitis cases not only become incurable but also act as a source of infection for other lactating animals in the herd [8]. In light of the aforementioned concerns, it is necessary to develop alternative methods to control mycotic mastitis therapy [12].

Essential oils (EO) could be an interesting natural alternative to prevent teat infections and to limit antibiotic use in mastitis treatment [15]. In addition, EOs are generally considered safe, and unlike antibiotics, bacteria do not appear to develop resistance to them, even during prolonged use [16,17]. As current treatment options become increasingly ineffective against resistant bacteria, the development of alternative phytotherapy products can combat antimicrobial resistance and provide sustainable animal health solutions that ensure safe and high-quality food production [18]. Natural products are an excellent solution as they are environmentally friendly and safe and are therefore highly valued in both organic and conventional farming [3,19]. Some EOs have biologically active compounds, such as thymol and carvacrol, that are responsible for their antimicrobial properties [20]. Hence, alternative EO treatment has been assessed in vitro on the major bacterial [21,22,23,24,25,26,27,28] and fungal mastitis pathogens [12,16,29]. Moreover, including *Candida* in studies investigating natural products for the treatment of bovine mastitis is important to assess their potential efficacy against this fungal pathogen and broaden understanding of therapeutic alternatives for managing mastitis cases caused by diverse microbial agents [30].

To the best of our knowledge, in vivo evaluation of the antifungal potential of EO-based formulations in bovine mastitis treatment has not been conducted yet. Therefore, the current research will focus on in vivo testing of EO-based formulations for possible use as an alternative treatment during lactation against mastitis-associated yeasts, giving clinical evidence on the expansion of pharmacological activity of this pharmaceutical formulation. Additionally, the prevalence of yeast present in the milk samples obtained from cows diagnosed with the subclinical or clinical form of mastitis will be assessed.

## 2. Results

### 2.1. Prevalence of Mastitis-Associated Pathogens and In Vitro Antimicrobial Activity of Phyto-Bomat EOs

**Figure 1:**
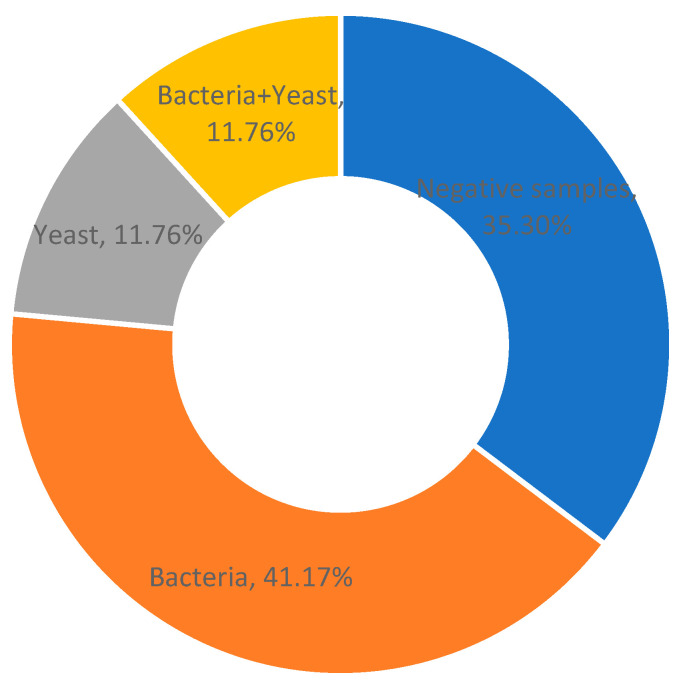
Prevalence of mastitis-associated pathogens.

Microbiological examination of 68 milk samples from cows diagnosed with the clinical and subclinical form of mastitis revealed the presence of microorganisms in 44 samples (64.70%): monoinfection with bacteria in 28 samples (41.17%), dual infection with bacteria and yeasts in 8 samples (11.76%), and monoinfection with yeasts in 8 (11.76%) samples. Twenty-four (35.30%) samples were negative for the presence of microorganisms, while in ten samples, more than one pathogen was isolated. These results are presented in Figure 1.

The most frequently isolated bacterial species belonged to the *Enterobacterales* family (*E. coli*, *Proteus mirabilis*, *Klebsiella* spp., and *Serratia marcescens*), with 66.67% of the isolates, followed by *Streptococcus* spp. (27.78%) (*S. dysgalactiae*, *S. uberis*) and *Staphylococcus* spp. (5.56%) (*S. aureus* and coagulase-negative staphylococci). Antimicrobial susceptibility testing of bacterial strains isolated from cows with mastitis is presented in Supplementary Materials Table S1. The antibacterial activity of Phyto-Bomat components was in the range of 1.56–6.25 mg/mL (Table 1). The MIC of Phyto-Bomat was in the range of 22.72–90.80 mg/mL and the MBC was from 45.40 to 181.60 mg/mL.

*Candida* spp. was isolated from all yeast-positive samples, with a low frequency of *C. albicans* (31.25%) compared to non-albicans species, which accounted for 68.75% of isolates: *C. glabrata* (37.50%), *C. parapsilosis* (18.75%), and *C. krusei* (12.50%). Antimicrobial susceptibility testing of yeast strains isolated from cows with mastitis and two laboratory control strains of yeast are presented in Supplementary Materials Table S2. The antibacterial activity of Phyto-Bomat individual EOs was in the range of 0.78–6.25 mg/mL (*Thymus vulgaris* L.), 0.39–6.25 mg/mL (*Thymus serpyllum* L.), 0.39–6.25 mg/mL (*Origanum vulgare* L.), and 0.78–12.5 mg/mL (*Satureja montana* L.) (Table 1). The MIC of Phyto-Bomat was in the range of 45.40–90.80 mg/mL and the MBC was from 90.80 to 181.60 mg/mL.

Although the Phyto-Bomat formulation is based exclusively on the combination of different essential oils, a type of pharmacological interaction between the antibiotic cephalexin and EOs was investigated in preclinical in vitro studies. The MICs of the individual Phyto-Bomat EOs were two to eight times higher than their MICs in combination with cephalexin. The FICI indices were in the range of 0.288–2.455. A synergistic effect (FICI ≤ 0.5) was observed in 18.6% of strains, mainly *Streptococcus* spp., and an additive effect (0.5 < FICI ≤ 1) was observed in 42.7% of strains. Indifferent interactions (1 < FICI ≤ 4) were observed in 38.7% of strains from the *Enterobacterales* family. None of the interactions were in the range of antagonism (FICI > 4). The synergism of EOs with antifungals has not been studied in vitro because most of the antifungals used in the treatment of mycotic mastitis, such as Amphotericin B, Nystatin, Itraconazole, Clotrimazole, Fluconazole, or Voriconazole, have a toxic effect on mammary tissue [12,14].

### 2.2. The Treatment Efficacy Regarding Isolate Status and Type of the Applied Treatments

**Figure 2:**
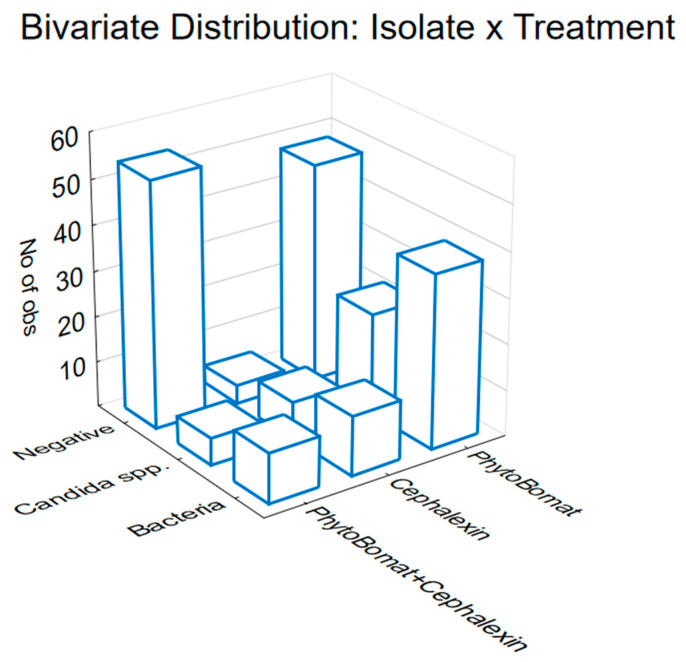
The prevalence of milk microbial contamination status (positive for bacteria, *Candida* spp., or negative) among studied animals in relation to the administered agent (Phyto-Bomat, cephalexin, and combination of the aforementioned), disregarding the time point of study in which the milk samples were obtained.

In order to understand the impact of different treatments on the occurrence of negative, bacteria, and Candida samples, the obtained results have demonstrated that, irrespective of the evaluated time point, there are statistically significant differences based on the type of treatment applied. This is confirmed by the Chi-square test results, which show a significant association (Chi-square = 31.03, df = 4, *p* = 0.00; Figure 2). The highest number of negative isolates was observed with the combination treatment, followed closely by Phyto-Bomat treatment. The combination of Phyto-Bomat and cephalexin resulted in a moderate reduction in bacterial isolates, suggesting a synergistic effect. Phyto-Bomat and cephalexin alone also reduced bacterial counts, with cephalexin showing a slightly greater effect than Phyto-Bomat.

### 2.3. The Treatment Efficacy Regarding Isolate Status and Observed Time Point

**Figure 3:**
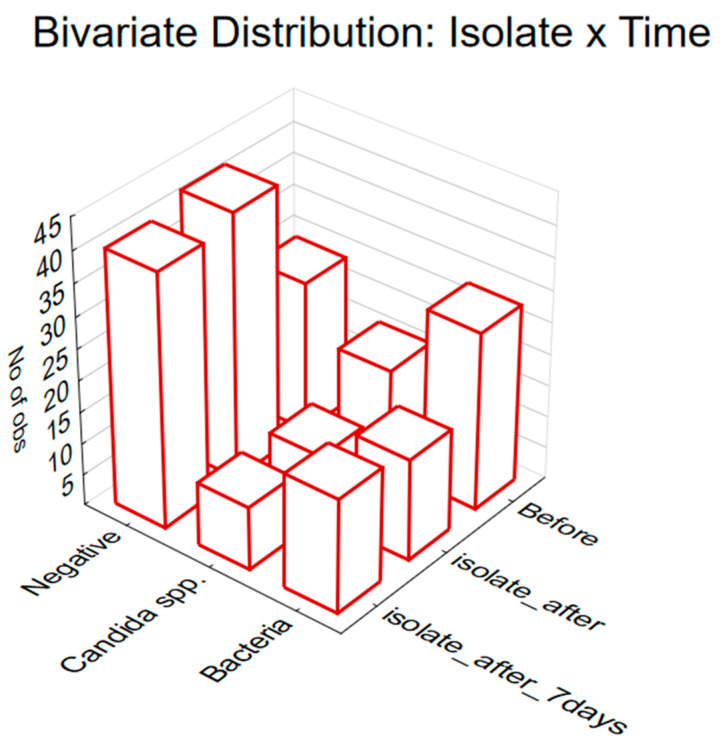
The prevalence of milk microbial contamination (presence of bacteria, *Candida* spp., and negative results) in studied animals before start of applied treatment (denoted as: before), one day after the end of treatment (denoted as: isolate_after), and seven days after the end of therapy (denoted as: isolate_after_7days).

In order to evaluate the impact of different time points on the occurrence of negative, bacteria, and *Candida* samples, the obtained results have demonstrated that, regardless of the type of applied treatment, there are statistically significant differences related to the evaluated time point. This is supported by the Chi-square test results, indicating a significant association (Chi-square = 11.51, df = 4, *p* = 0.02; Figure 3). Moreover, when focusing on the presence of Candida, a significant reduction has been observed in the group of cows treated solely with Phyto-Bomat after the treatment was applied, as illustrated in Figure 3.

## 3. Discussion

While mycotic mastitis is generally considered less common in dairy herds [1], some studies indicate a rising trend in the occurrence of this type of mastitis [31,32]. Conditions that elevate the risk of mycotic mastitis include the extended and unselective use of antibiotics and steroids in intramammary mastitis treatment. Additionally, fungal proliferation during antibiotic therapy is promoted by the elimination of antagonistic bacterial flora, reduced vitamin A levels in the glandular tissue, epithelial changes, and the irritating effects of antibiotics [32]. Furthermore, clinicians often overlook mycotic mastitis during the initial treatment, and administering antibiotics can worsen fungal mastitis, especially because certain antibiotics, such as penicillin and tetracycline, can serve as a nitrogen source for various fungal species [33]. To date, there have been no in vivo studies targeting mastitis caused by fungi. Hence, the development of a novel EO-based pharmaceutical formulation could help fill this research gap in mycotic mastitis treatment.

The results in the current study indicated that yeast infections make up an important portion of all udder infections in cows (11.76%). These findings align with scientific data showing that the prevalence of bovine mycotic mastitis varies between 2% and 13% [34,35]. While information regarding the prevalence of yeast on Serbian dairy farms is scarce, the current study’s results are relatively comparable with those obtained by Milanov et al. [6], where yeasts were isolated in 20 milk samples (6.02%). Furthermore, it has been reported that yeasts were more frequently isolated at the end of winter and in spring [35] and more frequently in small herds [33]. Nevertheless, it should be emphasized that, as highlighted by Zhou et al. [9], the presence of extensive production systems, environmental temperatures ranging 15–35 °C, and the duration of the disease represent significant risk factors contributing to the prevalence of mycotic mastitis. In addition, prior studies have identified several yeast species as potential causes of bovine mastitis, including *Candida*, *Aspergillus*, *Rhodotorula*, *Trichosporon*, *Saccharomyces*, and *Cryptococcus* [9,34,35]. On the other hand, the current study identified only *Candida* spp., confirming the prevailing understanding that *Candida* is the primary causative agent of yeast mastitis in dairy cows [7,9,34].

The increasing challenge of fungal disease treatment, including drug shortages, high treatment expenses, adverse side effects of the antifungal drugs, and the development of drug resistance or reduced drug efficacy against fungi, have increased focus among researchers of traditional medicinal approaches [12,36,37]. Moreover, addressing fungal infections in animals is more complex than tackling bacterial infections, as both animal and fungal cells share the characteristic of being eukaryotic [38,39]. Additionally, if the antifungal treatment targets a shared eukaryotic cell structure, it could potentially result in toxicity to animal cells, jeopardizing the safety of the host [38]. Furthermore, there are rising public concerns regarding the increased health and environmental risks linked to synthetic compounds. Consequently, there is a notable focus on exploring alternative, safe, and naturally derived approaches to develop new antifungal agents [39].

Taking into consideration the previously mentioned limitations of mycotic bovine mastitis therapy, EOs can represent one of the most promising natural products for fungal inhibition. Many EOs have demonstrated antifungal properties against a wide range of fungal species [40,41,42,43,44], as well as against mastitis-associated pathogens [12,15,16,45,46,47]. The EOs tested in our study showed notable antifungal potential in vitro (Table 1). Considerable antifungal properties of different EOs were reported by several authors [15,40,41,48,49,50]. On the contrary, Abd El Tawab et al. [51], who investigated the antifungal activity of seven EOs, reported resistance of *C. albicans* isolates to all of tested EOs. Interestingly, in the study conducted by Rocha et al. [52] *C. albicans* was resistant to oregano EO, leading to atypical colony sizes, possibly interfering with its pathogenicity. Despite the unchanged total cell count, there was a notable decrease in whole-cell concentration and growth of non-intact cells, possibly influenced by the oil’s compounds. However, it must be highlighted that the variability in antifungal potential could be related to seasonal and geographic factors [15]. When discussing the effectiveness of EOs, it is important to consider their biological source (plant species) and the composition of the oil [53]. Compounds present in the intramammary EO-based pharmaceutical formulation (Phyto-Bomat) used in the current study are well documented for antimicrobial potential against mastitis-associated pathogens [54,55,56,57]. Selected EOs have been previously tested in vitro (chemical composition, antimicrobial and antioxidant potential) [25,26]. Moreover, the significant antioxidative and antibacterial properties were utilized in the creation of a pharmaceutical formulation, offering an alternative to traditional mastitis treatment. Considering the well-documented fungicidal properties of common thyme, wild thyme, oregano, and mountain savory in the scientific literature, and recognizing the limited treatment options available for fungal mastitis, it becomes imperative to explore the in vivo anti-*Candida* effects of EOs. Therefore, the potential therapeutic application of the pharmaceutical formulation containing EOs for the therapy of mycotic bovine mastitis in lactating cows was chosen to be studied in the current research. Furthermore, in vitro [58], as well as in vivo [59], antimicrobial activity of the proposed EO-based formulation (Phyto-Bomat) against mastitis-associated bacteria has been proven.

Although many studies have assessed the effectiveness of EOs in treating bovine mastitis in laboratory conditions, only a few have focused on their in vivo efficacy. A major challenge in clinical trials of herbal medicines is determining if different products, extracts, or even batches of the same extract are comparable and equivalent [60]. Standardizing these preparations is difficult because they are complex mixtures of multiple phytochemicals. Moreover, the specific functions of many phytochemicals and their interactions with other molecules are still largely unknown, complicating the standardization process [60]. Hase et al. [61] investigated the therapeutic efficacy of a plant-based spray and gel, finding that phytotherapy enhances the udder’s immune capabilities. This treatment can suppress udder infections in subclinical mastitis without causing adverse effects. Additionally, an oregano EO-based ointment shows potential as an alternative to antibiotics for controlling subclinical mastitis caused by *S. aureus* and/or *E. coli* [62]. Interesting results were obtained in studies where two commercial EO-based products (Cinnatube^®^ and PhytoMast^®^) were tested on cows diagnosed with mastitis. Both products were administered intramammarily: Cinnatube^®^ for dry cows [63] and PhytoMast^®^ for lactating cows [64]. Both studies reported that these products were well tolerated, with no visible signs of udder irritation after use.

The results obtained in the current study indicate notable differences in the occurrence of *Candida* samples depending on the treatment applied, with the most remarkable distinction observed in cows subjected to a combined therapy (involving Phyto-Bomat and antibiotics). Furthermore, there is an increase in the number of negative results, indicating a reduction in microbial contamination immediately after treatment. Both *Candida* spp. and bacteria show a decrease in prevalence compared to the pre-treatment period, suggesting the treatment’s immediate effectiveness in reducing these microorganisms. Candida samples decreased immediately following the treatment with Phyto-Bomat and maintained this low level after 7 days, indicating the stability of Phyto-Bomat’s antifungal effect over this period. Additionally, Phyto-Bomat demonstrated a similar sustained activity against bacteria, with reduced levels observed both one day and seven days post-treatment. The antimicrobial potential of Phyto-Bomat could be attributed to its main components (i.e., terpenoids, specifically thymol and carvacrol) [58]. Terpenoids are one of the most valuable classes of natural origin compounds that are usually responsible for antimicrobial activity against disease-causing bacteria [37]. Along with this, the antifungal activity of Phyto-Bomat might be attributed to the nature of terpenoids, that—due to their high lipophilicity and low molecular weight—are capable of disrupting the cell membrane, causing cell death or inhibiting the sporulation and germination of fungi [37,38]. The literature data indicate that terpenoids show ineffective antimicrobial activity when used as singular compounds compared to the whole EO [38]. Moreover, due to high abundance of carvacrol and thymol in common thyme, wild thyme, oregano, and mountain savory, EOs have demonstrated substantial inhibitory effects against fungal pathogens by disrupting the integrity of the fungal cell membranes. The current study’s results support the possibility of an expansion of pharmacological activity of EO-based pharmaceutical formulations (Phyto-Bomat) through clinical evidence. Actually, the in vivo antifungal potential of Phyto-Bomat was proven through the significant *Candida* reduction after applied treatment. This could be explained by the fact that oregano, in particular, has been observed to impact spore germination and disrupt the membranes of fungal cells [37]. Furthermore, several authors have already described a strong antifungal activity of carvacrol and thymol against pathogenic strains of *C. albicans* isolated from the milk of cows with clinical mastitis [15,47,65]. Moreover, using antifungal drugs in combination with EOs is an effective approach to boost the efficacy of antifungal medications [66,67,68]. Furthermore, encouraging results were reported when using oregano and thyme EOs to enhance Itraconazole’s efficacy against azole *Cryptococcus neoformans* strains, whether they are susceptible or resistant to azole drugs [69]. The results obtained in the study by Carbone et al. [70] showed an increase in antifungal activity of nanoparticles loaded with Clotrimazole prepared with lavender or rosemary essential oil against local candidiasis.

Since EOs are complex mixtures of highly diverse secondary metabolites, they represent a rich resource of biologically active compounds that could be explored as potential candidates acting synergistically with antibiotics [71]. Therefore, EOs and their components could be used as additional therapy to antimicrobial drugs in order to reduce the development of resistance against antibacterial, antifungal, and antiviral drugs [72]. Additionally, the current study results have shown that the combination of Phyto-Bomat and antibiotic therapy have had important positive outcomes, indicating a strong synergistic effect. Moreover, synergism has the potential to lower the required dosage of a single drug, increasing its effectiveness while simultaneously reducing drug-related toxicity [68]. Co-treatment by EOs and antibiotics is expected to create a powerful antimicrobial effect that targets multiple aspects, helping to reduce or reverse antimicrobial resistance [73]. In future, this finding could be used to reduce the outbreak of secondary fungal infections in mastitis, and it underscores the importance of reducing antibiotic usage to prevent the emergence of secondary fungal mastitis. Additionally, this finding suggests that Phyto-Bomat may be beneficial as an alternative to conventional antimicrobial therapy, as well as an antifungal as added value. In addition, the use of this type of phytotherapy could bring considerable economic benefits, especially with regard to subclinical mastitis, which is responsible for most financial losses and could have great potential for application not only on conventional but also on organic farms.

The major novelty of this research is the environmentally friendly approach to bovine mastitis treatment through the use of Phyto-Bomat, an essential oil-based formulation. This green and sustainable method utilizes natural plant extracts that are in line with organic farming principles and reduces the reliance on synthetic antimicrobials. This study demonstrates the broad-spectrum antimicrobial activity of Phyto-Bomat and offers a safe and effective alternative that supports sustainable agriculture and combats antimicrobial resistance. Hence, further research is needed to explore how Phyto-Bomat can be integrated into veterinary practice as part of prevention programs against fungal infections in dairy cows due to its already proven antifungal, antibacterial, and antiviral properties.

## 4. Materials and Methods

### 4.1. Development of Intramammary EO-Based Formulation (Phyto-Bomat)

EO-based intramammary formulation (Phyto-Bomat) was based on four different EOs with previously proven in vitro [58] and in vivo [59] antibacterial activity in cows with diagnosed mastitis. This EO mixture consisted of common thyme (*Thymus vulgaris* L.), wild thyme (*Thymus serpyllum* L.), oregano (*Origanum vulgare* L.), and mountain savory (*Satureja montana* L). The obtained EO mixture was further diluted with common marigold (*Calendula officinalis* L.) and St. John’s wort (*Hypericum perforatum* L.) oil macerates (herbal drug:sunflower oil, 1:5) to a maximum volume of 15 mL for use in an intramammary injector. The assessed EOs of common thyme, wild thyme, oregano, and winter savory are available in the Serbian market from Pharmanais d.o.o., a certified Serbian manufacturer. Raw plant material (*Thymi folium*, *Serpylli herba*, *Origani herba*, and *Saturejae herba*) was sampled pre-distillation and verified for identity. Voucher specimens were deposited at the Herbarium of Drugs, Pharmacognosy and Phytotherapy Laboratory, Department of Pharmacy, Faculty of Medicine, University of Novi Sad.

The chemical composition and antimicrobial activity of each selected EO against bovine mastitis-associated pathogens were previously reported by Kovačević et al. [25,26]. Actually, the concentrations of EOs in the proposed formulation (Phyto-Bomat) were determined based on their minimum bactericidal concentration (MBC) values against the most prevalent mastitis-associated pathogens. The most dominant compounds among the EO components present in the proposed formulation were thymol and carvacrol [58].

### 4.2. Therapeutic Protocol

The experimental protocol was approved by the Animal Ethics Committee of the Ministry of Agriculture, Forestry and Water Management-Veterinary Directorate (9000-689/2, 6 July 2020). In this study, a total of 68 animals with diagnosed mastitis were selected for in vivo testing, from two Holstein–Friesian dairy farms located in the Province of Vojvodina, Serbia, from May to July. The study involved Holstein–Friesian cows, aged 2 to 5 years, in different stages of the lactation phase, housed in a free-stall system within a commercial dairy farm. On the farm, cows were fed twice daily using total mixed ration with unrestricted access to water. The clinical form of mastitis was assessed by veterinarians performing clinical examination, while the subclinical form was confirmed by the California Mastitis Test using somatic cell count in the milk samples. Clinical signs of mastitis included symptoms such as swelling, pain, and redness. Alterations in the first milk streams, such as clot formation and changes in color and density, were present. The animals were divided into three experimental groups. The first group of cows was treated with conventional antibiotic treatment (n = 25), the second one was treated with Phyto-Bomat (108), while the third group underwent a combination therapy involving both Phyto-Bomat and antibiotics (71). For the conventional antibiotic treatment, cows were treated with the commercially available cephalexin [74], as per the manufacturer’s recommendations, following an antibiotic susceptibility test conducted according to the farm’s treatment protocol. Phyto-Bomat was applied intramammarily, twice daily after the morning and evening milking, for 5 consecutive days. Cows treated with the combination were initially treated with cephalexin [74] administered intramuscularly, in accordance with the manufacturer’s recommendations, followed by the application of Phyto-Bomat twice a day for 5 consecutive days. The treatment efficacy was evaluated by microbiological examination, taking milk samples from each animal before the treatment, one day and seven days after the end of the therapy.

### 4.3. Isolation and Identification of the Pathogens and In Vitro Studies of EO Antimicrobial Activity

Cows were hand-milked twice daily, in the morning and evening. Samples for microbiological examination were collected during the morning milking. Prior to milking, the udder and teats were washed with water, and the teats were then disinfected and dried with tissue paper. The tip of each teat was disinfected using a 70% ethanol, after which milk samples were taken. These samples were taken using sterile gloves and placed in sterile bottles. The first few streams of milk were discarded and approximately 10 mL of milk was collected in labeled sterile tubes and stored in an ice container at 4 °C during transport to the Laboratory for testing biological material, food, and animal feed, “In vitro LAB” DOO Šabac, Republic of Serbia. Standard bacteriological diagnostic methods were employed for bacteriological isolation, determination, and identification of mastitis-causing pathogens [25,26]. Yeast strains were cultured on Sabouraud dextrose agar plates, which were then incubated at 30 °C for 48 h. Isolates were subsequently identified, using the “API 20 C AUX” (bioMérieux, Marcy-l’Etoile, France).

Antibacterial activity of EO mixture and its individual components *T. vulgaris* L., *T. serpyllum* L., *O. vulgare* L., and *S. montana* L. was investigated by broth microdilution method in accordance with previously described protocols [25,26].

Antifungal activity was tested by broth microdilution method against clinical isolates and two ATCC laboratory control strains of yeasts (KWIK-STIK™, Microbiologics, Saint Cloud, MN, USA): *Candida albicans* ATCC 24433 and *C. krusei* ATCC 6258. Lyophilized microorganisms were hydrated before experiments, inoculated onto Sabouraud dextrose agar/broth (SDA/SDB, Oxoid Ltd., Basingstoke, Hampshire, UK), and cultivated in aerobic conditions for 24–48 h at 35 ± 1 °C. The suspension of microorganisms used as inoculum was prepared from fresh cultures in a saline solution to a density of 0.5 per McFarland standard (bioMérieux, Marcy-l’Etoile, France).

The minimum inhibitory concentrations (MICs) were determined by broth microdilution test in flat-bottomed 96-well microtiter plates according to the European Committee for Antimicrobial Susceptibility Testing [75,76]. EOs were prepared in fresh SDB, in concentrations ranging from 0.048 to 12.50 mg/mL; each concentration was set in duplicate and inoculated with 1 × 10^4^ CFU/mL of yeasts. For detection of cell growth and metabolism, broths were supplemented with redox indicator resazurin (7-Hydroxy-3H-phenoxazin-3-one 10-oxide) (Sigma-Aldrich-Merck KGaA, Darmstadt, Germany), which is a blue non-fluorescent dye that is reduced to pink metabolite resorufin (7-Hydroxy-3H-phenoxazin-3-one) if microorganisms are viable. MICs of samples were determined after the incubation for 20–48 h at 35 °C in aerobic conditions as the lowest concentration that inhibited the growth of yeasts. Minimum fungicidal concentration (MFC) was determined after subcultivation of all wells without visible growth of yeasts onto SDA and incubation for additional 20–48 h at 35 °C in aerobic conditions. Positive controls (microorganisms in medium) and negative controls (only medium with EOs) were included in experiments. Each test was repeated three times.

The type of the pharmacological interaction (synergism, additive effect, indifferent effect, antagonism) between each Phyto-Bomat EO and the antibiotic cephalexin was investigated using the checkerboard method [77]. The synergistic effect of the EO–cephalexin combination was investigated at a concentration above the MIC (2 × MIC) and at four concentrations below the MIC (1/2–1/16) of the individual compounds. The effect was estimated by calculating the fractional inhibitory concentration (FIC) and the fractional inhibitory concentration indices (FICIs). The FIC of each compound was calculated by dividing the concentration of the compound in the effective MIC of the combination, by the MIC of the compound alone (e.g., FIC_EO_ = MIC_EO-cephalexin combination_/MIC_EO_). The FICI values were calculated as the sum of the FIC_EO_ and FIC_cephalexin_ and interpreted as follows: FICI ≤ 0.5 synergy; 0.5 < FICI ≤ 1 additive effect; 1 < FICI ≤ 4 indifference (no interaction) and FICI > 4.0 antagonism [78].

### 4.4. Data Analysis

The obtained data were built into a dataset by application of Microsoft Office Excel and statistically analyzed by Tibco Statistica^®^ software v. 13.5 (TIBCO Software Inc. TIBCO Statistica™ 13.5.0. 2018. TIBCO^®^ Data Science—Statistica™ 13.5.0). Data were evaluated by descriptive statistics, while differences between evaluated categorical variables regarding the frequency of obtained results were evaluated by application of Chi-square test, whereas the level of significance was kept at *p* = 0.05.

## Figures and Tables

**Table 1:** Minimum inhibitory (MIC), bactericidal (MBC), and fungicidal (MFC) concentrations of Phyto-Bomat individual EOs *Thymus vulgaris* L., *Thymus serpyllum* L., *Origanum vulgare* L., and *Satureja montana* L.

Microorganisms	TV (mg/mL)		TS (mg/mL)		OV (mg/mL)		SM (mg/mL)	
Bacteria	MIC	MBC	MIC	MBC	MIC	MBC	MIC	MBC
*Streptococcus* spp.	3.12–6.25	6.25–12.5	0.39–3.12	0.78–12.5	3.12–6.25	6.25–12.5	0.39–6.25	0.78->12.5
*Staphylococcus* spp.	6.25	12.5	1.56–3.12	3.12–6.25	3.12	6.25	6.25	12.5
*Enterobacterales*	1.56–3.12	3.12–6.25	1.56–3.12	3.12–6.25	0.78–3.12	1.56–6.25	3.12	6.25
Yeasts	MIC	MFC	MIC	MFC	MIC	MFC	MIC	MFC
*C. albicans*	1.56–3.12	3.12–6.25	0.78–3.12	3.12–12.5	1.56–3.12	3.12–6.25	1.56–3.12	3.12–6.25
*C. glabrata*	1.56–6.25	3.12–12.5	0.78–3.12	1.56–6.25	0.78–1.56	1.56–3.12	3.12–6.25	6.25–12.5
*C. parapsilosis*	6.25	12.5	1.56	3.12	1.56	3.12	6.25	12.5
*C. krusei*	3.12	6.25	1.56	3.12	1.56	6.25	6.25	12.5
*Candida albicans* ATCC 24433	0.78	1.56	0.39	0.78	0.39	0.78	0.78	1.56
*C. krusei* ATCC 6258	1.56	3.12	0.78	3.12	0.78	3.12	1.56	3.12

TV—*Thymus vulgaris* L.; TS—*Thymus serpyllum* L.; OV—*Origanum vulgare* L.; SM—*Satureja montana* L. MIC—minimum inhibitory concentration; MBC—minimum bactericidal concentration; MFC—minimum fungicidal concentration.

## Data Availability

The data used to support the findings of this study are available in the present manuscript.

## Supplementary Materials

The following supporting information can be downloaded at: https://www.mdpi.com/article/10.3390/antibiotics13070575/s1. Supplementary Table S1: Antimicrobial susceptibility testing of bacterial strains isolated from cows with mastitis; Supplementary Table S2: Antimicrobial susceptibility testing of yeast strains isolated from cows with mastitis and two laboratory control strains of yeast.
