# Supplementary material for: Clinical Evidence on Expansion of Essential Oil-Based Formulation’s Pharmacological Activity in Bovine Mastitis Treatment: Antifungal Potential as Added Value
Source: Antibiotics (Basel). 2024 Jun 22;13(7):575. doi: 10.3390/antibiotics13070575 (PMC11273906; doi:10.3390/antibiotics13070575)
Supplement: Supplementary file 1 [file antibiotics-13-00575-s001.zip › antibiotics-3004592-supplementary.pdf]

Supplementary Table S1. Antimicrobial susceptibility testing of bacterial strains isolated from cows with mastitis.

Bacterial Strain	AMP	AMX	CRO	AMC	CLO	ERY	LIN	GEN	NEO	TET	ENR	NB	SXT
1. *E. coli*	R	R	R	R	R	R	R	S	S	R	S	R	S
2. *E. coli*	R	R	R	R	R	R	R	S	S	I	S	R	S
3. *E. coli*	R	R	R	R	R	R	R	S	S	I	S	R	S
4. *E. coli*	R	R	R	R	R	R	R	S	S	R	S	R	S
5. *E. coli*	R	I	S	S	R	R	R	S	S	I	S	R	S
6. *E. coli*	R	I	S	S	R	R	R	S	S	S	S	R	S
7. *E. coli*	R	R	R	R	R	R	R	S	S	I	S	R	S
8. *E. coli*	R	I	R	R	R	R	R	S	S	S	S	R	S
9. *E. coli*	R	I	R	R	R	R	R	S	S	I	S	R	S
10. *E. coli*	R	S	S	S	R	R	R	S	S	I	S	R	S
11. *P. mirabilis*	R	R	S	S	R	S	R	S	S	R	S	R	S
12. *P. mirabilis*	R	R	S	S	R	R	R	S	S	R	S	R	I
13. *P. mirabilis*	R	R	S	R	R	R	R	S	S	R	S	R	S
14. *P. mirabilis*	R	R	S	S	R	R	R	S	S	R	S	R	S
15. *P. mirabilis*	R	R	S	R	R	R	R	S	S	R	S	R	S
16. *S. marcescens*	R	R	S	R	R	R	R	S	S	I	S	R	S
17. *S. marcescens*	R	R	S	R	R	R	R	S	S	R	S	R	S
18. *S. marcescens*	R	R	S	R	R	R	R	S	S	R	S	R	S
19. *S. marcescens*	R	R	S	R	R	R	R	S	S	R	S	R	S
20. *S. marcescens*	R	R	S	R	R	R	R	S	S	R	S	R	S
21. *S. marcescens*	R	R	S	R	R	R	R	S	S	R	S	R	S
22. *S. marcescens*	R	R	S	R	R	R	R	S	S	I	S	R	S
23. *K. oxytoca*	R	R	S	R	R	R	R	S	S	R	S	R	S
24. *K. oxytoca*	R	R	R	R	R	R	R	S	S	R	S	R	S
25. *Streptococcus*	R	S	R	S	R	I	R	I	R	R	S	I	R
26. *Streptococcus*	R	S	R	S	R	I	R	I	R	R	S	I	R
27. *Streptococcus*	S	S	I	S	R	I	R	S	R	S	S	R	R
28. *Streptococcus*	S	S	S	S	R	S	S	R	R	S	S	S	R
29. *Streptococcus*	R	R	R	R	R	R	R	I	R	R	R	R	R
30. *Streptococcus*	R	R	R	R	R	R	R	I	R	R	R	R	R
31. *Streptococcus*	R	R	R	R	R	I	R	S	R	R	R	R	R
32. *Streptococcus*	R	I	S	S	R	R	R	S	S	I	S	I	S
33. *Streptococcus*	R	R	S	S	R	R	R	S	S	R	S	R	R
34. *Streptococcus*	R	R	I	S	R	R	I	S	R	R	S	R	R
35. *S. aureus*	R	I	S	S	R	S	S	S	S	S	S	S	S
36. *CNS*	S	S	I	S	R	S	S	S	S	S	S	S	S

Beta lactams: AMP, ampicillin; AMX, amoxycillin; CRO, ceftriaxone; AMC, amoxicillin/clavulanic acid; CLO, cloxacillin; macrolide ERY, erythromycin; lincosamide LIN, lincomycin; aminoglycosides: GEN, gentamicin; NEO, neomycin; TET, tetracycline; fluoroquinolone ENR, enrofloxacin; NB, novobiocin; SXT, trimethoprim/sulfamethoxazole. (S-sensitive: I-intermediate, R-resistant); CNS-coagulase negative *Staphylococcus.*

Supplementary Table S2. Antimicrobial susceptibility testing of yeast strains isolated from cows with mastitis, and two laboratory control strains of yeast.

Yeast Strain	AMB	FLU	ITR	VOR
1. *C. albicans*	I	S	S	S
2. *C. albicans*	S	R	I	S
3. *C. albicans*	S	S	I	S
4. *C. albicans*	I	R	I	S
5. *C. albicans*	R	S	I	S
6. *C. glabrata*	I	I	R	S
7. *C. glabrata*	S	I	R	S
8. *C. glabrata*	R	I	R	S
9. *C. glabrata*	R	I	I	S
10. *C. glabrata*	R	I	R	S
11. *C. glabrata*	R	I	I	S
12. *C. parapsilosis*	S	R	I	S
13. *C. parapsilosis*	S	R	R	S
14. *C. parapsilosis*	I	S	I	S
15. *C. krusei*	S	R	R	S
16. *C. krusei*	S	S	R	S
17. *C. albicans* ATCC 24433	S	I	S	S
18. *C. krusei* ATCC 6258	S	I	I	S

AMB—Amphotericin B; FLU—Fluconazole; ITR—Itraconazole; VOR—Voriconazole (S-sensitive: I-intermediate, R-resistant)
